# Association between *HRAS* rs12628 and rs112587690 polymorphisms with the risk of melanoma in the North American population

**DOI:** 10.1007/s12032-012-0255-3

**Published:** 2012-05-22

**Authors:** Sara Tomei, Sharon Adams, Lorenzo Uccellini, Davide Bedognetti, Valeria De Giorgi, Narnygerel Erdenebileg, Maria Libera Ascierto, Jennifer Reinboth, Qiuzhen Liu, Generoso Bevilacqua, Ena Wang, Chiara Mazzanti, Francesco M. Marincola

**Affiliations:** 1 Division of Surgical, Molecular, and Ultrastructural Pathology, Section of Molecular Pathology, University of Pisa and Pisa University Hospital, 56100 Pisa, Italy; 2 Infectious Disease and Immunogenetics Section (IDIS), Department of Transfusion Medicine, National Institutes of Health, Clinical Center and trans-NIH Center for Human Immunology (CHI), 10 Center Drive, Bethesda, MD 20892 USA; 3 HLA Laboratory, Department of Transfusion Medicine, National Institutes of Health, Clinical Center, Bethesda, MD USA; 4 Institute of Infectious and Tropical Diseases, University of Milan, L. Sacco Hospital, Milan, Italy; 5 Center of Excellence for Biomedical Research (CEBR), University of Genoa, Genoa, Italy; 6 National Cancer Institute, “Fondazione G Pascale”, via G. Semmola, Naples, Italy; 7 Department of Biochemistry, Biocenter, University of Wuerzburg, Wuerzburg, Germany; 8 Genelux Corporation, San Diego Science Center, San Diego, CA USA

**Keywords:** *HRAS*, Polymorphism, Melanoma, rs12628, rs112587690

## Abstract

*HRAS* belongs to the *RAS* genes superfamily. *RAS* genes are important players in several human tumors and the single-nucleotide polymorphism rs12628 has been shown to contribute to the risk of bladder, colon, gastrointestinal, oral, and thyroid carcinoma. We hypothesized that this SNP may affect the risk of cutaneous melanoma as well. *HRAS* gene contains a polymorphic region (rs112587690), a repeated hexanucleotide -GGGCCT- located in intron 1. Three alleles of this region, P1, P2, and P3, have been identified that contain two, three, and four repeats of the hexanucleotide, respectively. We investigated the clinical impact of these polymorphisms in a case–control study. A total of 141 melanoma patients and 118 healthy donors from the North America Caucasian population were screened for rs12628 and rs112587690 polymorphisms. Genotypes were assessed by capillary sequencing or fragment analysis, respectively, and rs12628 CC and rs112587690 P1P1 genotypes significantly associated with increased melanoma risk (OR = 3.83, *p* = 0.003; OR = 11.3, *p* = 0.033, respectively), while rs112587690 P1P3 frequency resulted significantly higher in the control group (OR = 0.5, *p* = 0.017). These results suggest that rs12628 C homozygosis may be considered a potential risk factor for melanoma development in the North American population possibly through the linkage to rs112587690.

## Introduction

Cutaneous malignant melanoma is the most aggressive common skin tumor. It is characterized by a multifactorial etiology. Sun exposure and genetic susceptibility have been proposed as major etiological and predisposing factors [1–3].

Mammalian cells contain three functional *RAS* proto-oncogenes, known as *HRAS*, *KRAS,* and *NRAS*, which encode small GTP-binding proteins. *RAS* genes have been characterized as major participants in the development and progression of a series of human tumors, such as gastrointestinal cancer, lung cancer, thyroid cancer, melanoma, and breast cancer. It was reported that just one point mutation occurring in codons 12, 13, or 61 and rarely in cod0on 146 could result in continuous stimulation of cell proliferation; thus, codons 12, 13, 61, and 146 are also called mutation hotspots. Alternatively, a 5- to 50-fold amplification of the wild-type gene results in over-expression and constitutive hyper-activation of the gene [4, 5]. These alterations increase the proportion of GTP-bound *HRAS* protein independently of external signals [6–8].

Besides the mutation hotspots, inherited polymorphisms in the *HRAS* sequence were described [9, 10]. A single-nucleotide polymorphism at *HRAS* cDNA position 81T > C (rs12628), originally described by Taparowsky et al. [11], was shown to be associated with the risk of human cancers. It is present in codon 27 of exon 1 of *HRAS*, which is located in a wobble base position. Recently, it was demonstrated that the C-allele of this SNP could increase the risk of oral carcinoma, colon, and gastric cancer [12–14]. rs12628 SNP has also been reported to play a significant role in predisposition to bladder cancer [12, 15]. However, very few studies have been conducted to examine the *HRAS* rs12628 polymorphism and to understand its role fully.

While sequencing 15 melanoma cell lines for genes belonging to the MAPK pathway [16], we found 7 cell lines carrying rs12628 CT genotype and 3 cell lines carrying rs12628 CC genotype. We then compared the rs12628 genotype and allele frequencies with the ones reported in the HapMap database and found that overall the frequencies of the C-allele and the CC genotype were higher in the 15 cell lines as compared with the ones reported in the HapMap project for the Caucasian populations. Since the functional and epidemiological relevance of rs12628 is unknown, we decided to perform a case–control study to investigate its role in melanoma. Moreover, since a strong linkage disequilibrium between rs12628 and rs112587690 has been reported by Sol-Church et al. [17], we included rs112587690 screening in the study. Sequence analysis of the internal structure of rs112587690 showed that allele P1 consists of 2 perfect repetitions of a consensus sequence (GGGCCT), allele P2 displays 3 repetitions of the consensus, and allele P3 consists of three perfect (GGGCCT) and one imperfect (GGGGCT) repetitions of the consensus [18].

Here, we tested the association of rs12628 and rs112587690 with the risk of cutaneous melanoma.

## Materials and methods

### DNA samples

Germ line DNA was isolated from blood of 118 Caucasian healthy donors and 141 Caucasian melanoma patients with metastatic melanoma that underwent immunotherapy with high-dose IL-2 at the Surgery Branch, National Institutes of Health, Bethesda, MD. Patients and healthy controls were matched according to age and sex. Written informed consent was obtained from all subjects before testing. DNA was screened for rs12628 and rs112587690 polymorphisms by sequencing or fragment analysis, respectively.

### *HRAS* sequencing and fragment analysis

PCR for rs12628 polymorphism was performed in 20 μl final volume, containing 50 ng of genomic DNA, 10 μl of Qiagen HotStarTaq Master Mix Kit (Valencia, CA) and 1 μM of forward and reverse primers with the following cycling conditions: initial denaturation at 95 °C for 10 min; 35 cycles at 95 °C for 30 s, 56 °C for 30 s and 72 °C for 30 s; final step 72 °C for 10 min. Primers were selected using Primer3 software:

- *HRAS*_rs12628_F: GTGGGTTTGCCTTCAGA
- *HRAS*_rs12628_R: CTATCCTGGCTGTGTCCTG

PCR products were then sequenced on Applied Biosystems 3730 Genetic Analyzer (Foster City, CA) and analyzed by Sequencher software (Genecodes, Ann Arbor, MI).

PCR for rs112587690 SNP was performed in 20 μl final volume, containing 100 ng of genomic DNA, 10 μl of Qiagen HotStarTaq Master Mix Kit (Valencia, CA), and 0.5 μM of forward and reverse primers with the following cycling conditions: initial denaturation at 95 °C for 10 min; 5 cycles at 95 °C for 30 s, 57 °C for 30 s and 72 °C for 30 s; then 35 cycles at 95 °C for 30 s, 55 °C for 30 s and 72 °C for 30 s; final step 72 °C for 10 min. Primers were selected using Primer3 software:

- *HRAS*_rs112587690_F (6-FAM labeled): GTGGGTTTGCCCTTCAGAT
- *HRAS*_rs112587690_R: ATATTCCGTCATCGCTCCTC

PCR products were previously sequenced on Applied Biosystems 3730 Genetic Analyzer (Foster City, CA) to confirm gene sequence and then diluted 1:100. One microliter of diluted PCR products was run on Applied Biosystems 3730 Genetic Analyzer (Foster City, CA) for fragment analysis, using 0.3 μl of GeneScan 600 LIZ Size Standard (Applied Biosystems, Carlsbad, CA) for each reaction.

Each PCR used distilled water instead of DNA as a negative control.

DNA fragments were finally analyzed using Gene Mapper software (Applied Biosystems). The size of the products were P1 (145 bp), P2 (151 bp) and P3 (157 bp).

### Gene expression data

Total RNA was isolated from 112 metastatic tumor samples from as many patients of the cases group. Tumor samples were from snap frozen biopsies.

cDNAs were fragmented, biotinylated, and hybridized to the GeneChip Human Gene 1.0 ST Arrays (Affymetrix WT Terminal Labeling Kit). Probe normalization, background correction, Log2 transformation and summarization were performed using Robust Multi-Chip Average (RMA). Gene summary was obtained by averaging the mean of the probe values end expressed as Log2 intensity [19].

*HRAS* mRNA expression values (log2 transformed) were extrapolated and evaluated according to rs12628 and rs112587690 genotypes.

### Statistical analysis

Hardy–Weinberg equilibrium was tested by Chi-square. Odds ratios (ORs) and 95 % confidence intervals (95 % CIs) were calculated. The haplotype analysis of rs12628 and rs112587690 polymorphisms was carried out using pharmgat online tool (https://pharmgat.org/). The association between the rs12628 and rs112587690 genotypes and *HRAS* mRNA expression was tested by one-way ANOVA and two-tailed student's *t* tests. All the statistical analyses were performed using Partek software and Instat 3 software.

Transcription factor binding site analysis of the intronic rs112587690 sequence was carried out using online TFSEARCH online software (http://www.cbrc.jp/research/db/TFSEARCH.html).

## Results

The distribution of rs12628 genotypes and allele frequency in cases and controls are shown in Table 1. In healthy controls, the frequencies of TT, TC, and CC genotypes were 47, 48, and 5 % which did not deviate from the Hardy–Weinberg equilibrium (Chi-square = 3.3, *p* = 0.07), while those frequencies were 46, 37, and 17 % in melanoma patients, respectively. A high frequency of “C” allele was found in cases compared with controls, but the difference in allele frequency was not statistically significant. The distribution of genotypes revealed a higher frequency of CC in cases compared with controls (*p* = 0.003).

**Table 1:** Genotypes and alleles distribution for rs12628 and rs112587690 polymorphisms

	Cases (frequency) *n* = 141	Controls (frequency) *n* = 118	Odds ratio (CI)	*p* value
rs12628				
Genotypes			CC vs CT + TT	
TT	65 (0.46)	55 (0.47)	3.83 (1.51–9.72)	0.003
TC	52 (0.37)	57 (0.48)		
CC	24 (0.17)	6 (0.05)		
Alleles			C vs T	
T	182 (0.64)	167 (0.71)	1.33 (0.92–1.93)	0.16
C	100 (0.36)	69 (0.29)		
rs112587690				
Genotypes			P1P3 vs all	
P1P1	6 (0.04)	0	0.5 (0.29–0.88)	0.017
P2P2	4 (0.03)	1 (0.01)		
P3P3	65 (0.46)	55 (0.47)	P1P1 vs all	
P1P2	14 (0.10)	5 (0.04)		
P1P3	29 (0.21)	40 (0.34)	11.3 (0.63–204)	0.033
P2P3	23 (0.16)	17 (0.14)		
Alleles			P2 vs (P1 + P3)	
P1	55 (0.19)	45 (0.19)	1.67 (0.99–2.85)	0.08
P2	45 (0.16)	24 (0.10)		
P3	182 (0.65)	167 (0.71)		

*CI* confidence interval

To evaluate the risk of cutaneous melanoma according to rs12628 genotypes, ORs and their 95 % confidence intervals were calculated. Compared with TC + TT genotypes, CC genotype significantly associated with a 3.83-fold increased risk (OR = 3.83, CI = 1.51–9.72, *p* = 0.003).

The rs112587690 genotype frequencies conformed the Hardy–Weinberg equilibrium in controls (Chi-square = 6.7, *p* = 0.08). The distribution of rs112587690 and its allele frequency are shown in Table 1. The allelic difference in cases and controls resulted not statistically significant, although there is a trend of P2 allele to be higher in cases than in controls (OR = 1.67, CI = 0.99–2.85, *p* = 0.08).

When analyzing genotype frequencies, the genotype P1P3 resulted significantly higher in controls than in cases (OR = 0.5, CI = 0.29–0.88, *p* = 0.017), while the genotype P1P1 significantly associates with cases with an 11-fold increased risk (OR = 11.3, CI = 0.63–204, *p* = 0.033).

We also examined the linkage between the rs12628 and rs112587690 polymorphisms in all 141 cases and 118 controls. Consistent with the literature [17], in every instance SNP rs12628 appeared in perfect linkage disequilibrium with the rs112587690 hexanucleotide polymorphism (*D*′ = 1). Thus, individuals harboring the rs12628 TT genotype predictably carried rs112587690 homozygous P3 alleles. Conversely, individuals with rs12628 CC genotype carried the rs112587690 minor allele P1 and/or P2 in any combination (P1, P2, or P1P2). Not surprisingly, heterozygous rs12628 CT individuals segregated into two distinct rs112587690 genotypes: P1P3 and P2P3.

**Fig. 1:**
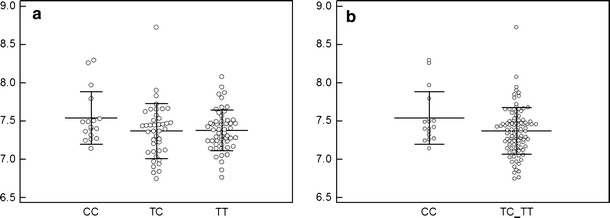
*HRAS* mRNA expression according to rs12628 genotypes. Log2 transformed *HRAS* expression values from microarray analysis of tumors evaluated according rs12628 CC, TC and TT genotypes separately (**a**) and CC versus TT + TC grouped genotypes (**b**)

We speculated that rs12628 and rs112587690 polymorphisms could have a potential influence on transcriptional regulation of *HRAS* gene, thus we sought to examine the level of *HRAS* mRNA in tumor tissues according to the different genotypes of rs12628 and rs112587690 polymorphisms. Toward this goal, we extrapolated the log2 intensity values from microarray analysis and evaluated the possible differential expression by one-way ANOVA and unpaired Student’s *t* test. The difference in *HRAS* mRNA expression level tended to be higher in metastases of patients carrying rs12628 CC genotype than in those carrying CT and TT genotypes, but this difference was not statistically significant (TT vs TC vs CC: *p* = 0.14; CC vs TC + TT: *p* = 0.08) (Fig. 1). No differential *HRAS* mRNA expression was observed according to the rs112587690 genotypes.

Different polymorphisms could affect the binding site sequences for several transcription factors. In order to investigate the possible biological meaning of rs112587690 polymorphism, TFBS (transcription factor binding site) prediction analysis of the polymorphic region was carried out by using online TFSEARCH program. Compared with P1 and P2 allele sequences, P3 allele sequence turned out to have an additional binding site for *Sp1* (Specific protein 1) and one for *ADR1* (alcohol dehydrogenase regulator 1).

## Discussion

Several molecular alterations in the *RAS* genes have been identified such as minisatellites and acquired mutations in various cancers including melanoma. In this study, we assessed germline variants of *HRAS* in the context of cutaneous melanoma; rs12628 was frequently observed and considered an informative SNP. Thus, we conducted a case–control polymorphic study of *HRAS* rs12628 to assess the role of this SNP in cancer.

Assuming a recessive genetic model of inheritance, a statistically significant result was found when comparing carriers of the homozygous mutant C-allele to heterozygotes CT and wild-type carriers TT (*p* = 0.003), with a 3-fold increased risk of melanoma in CC individuals. Thus, *HRAS* rs12628 represents a strong susceptibility factor for the development of melanoma. However, these epidemiological data do not reveal the mechanisms by which *HRAS* rs12628 SNP modifies melanoma risk. Acquired amino acid mutations in the hotspot codons 12, 13, 61, and 146 prolong the GTP-bound activated state of the *HRAS* product; in contrast, SNP rs12628 is located in a wobble base position, that should not lead to alterations in protein structure. It is conceivable that this probably nonfunctional polymorphism is linked to other polymorphic sites in functional intron regions of *HRAS*.

A potential linkage candidate is the hexanucleotide repeat located about 80 bp upstream of the 5′-end of exon 1 (rs112587690). Sol-Church et al. [17] observed strong linkage disequilibrium between rs12628 and rs112587690 polymorphisms. To confirm this data, we screened cases and controls also for rs112587690 polymorphism, we found an 11-fold increased risk of melanoma in homozygotes P1P1 and all the other genotypes (*p* = 0.033, OR = 11.3), while genotypes P1P3 resulted significantly associated with control group (OR = 0.5, *p* = 0.017). Thus, *HRAS* rs112587690 represents an additional susceptibility factor for the development of melanoma. Moreover, according to the report aforementioned [17], rs12628 and rs112587690 polymorphisms resulted to be in strong linkage disequilibrium (*D*′ = 1). The strong linkage disequilibrium observed between the two polymorphisms could reflect a differential transcriptional regulation of *HRAS* gene, according to the specific genotype.

We speculated that rs12628 and rs112587690 polymorphisms might have a potential influence on transcriptional regulation of *HRAS* gene, and thus we sought to examine the level of *HRAS* mRNA in the tumor tissues according to rs12628 and rs112587690 genotypes. Overall, melanoma metastases from patients carrying rs12628 CC genotype trended toward higher *HRAS* mRNA expression, followed by those from patients carrying TT and TC genotypes, respectively. However, these differences were not statistically significant (TT vs TC vs CC: *p* = 0.14; CC vs TC + TT: *p* = 0.08) (Fig. 1). Further studies are needed to evaluate whether *HRAS* mRNA expression is influenced by rs12628 genotypes in larger groups. No differential *HRAS* expression was found for rs112587690 polymorphism.

Whereas recognition of functional variants in coding regions is easy, detecting functional change in non-coding DNA is more difficult because there is no clear connection between nucleotide differences and function for these sequences, and regulatory regions can be located far from the coding region [20]. Nonetheless, it is important to understand how sequence change can influence the function of transcriptional regulation. We performed transcription factor binding sites prediction (in silico) using TFSEARCH program. An additional binding site for *Sp1* (Specific protein 1) and one for *ADR1* (alcohol dehydrogenase regulator 1) were predicted in the P3 allele sequence as compared to P1 and P2 alleles. Further functional studies on the role of these transcription factors are needed to better understand the importance of rs112587690 polymorphism as a regulator of *HRAS* transcription.

## Conclusion

This study provides evidence that the *HRAS* polymorphism rs12628 may be a risk factor for the development of melanoma in the Caucasian North American population possibly through the linkage to rs112587690 polymorphic site. Additional studies with large samples are needed to confirm these results.
